# Supplementary material for: Phenotyping 172 strawberry genotypes for water soaking reveals a close relationship with skin water permeance
Source: PeerJ. 2024 Aug 29;12:e17960. doi: 10.7717/peerj.17960 (PMC11366227; doi:10.7717/peerj.17960)
Supplement: Supplemental Information 2 [file peerj-12-17960-s002.docx]

**Table S1:**

**List of genotypes in each collection.**

| Population | Genotype (name/ code) | Population | Genotype (name/ code) | Population | Specie | Genotype (name/ code) |
| --- | --- | --- | --- | --- | --- | --- |
| F2 | CS-001 | cultivars | 141033 | species | *F. mandshurica* | Baegdusan |
| F2 | CS-002 | cultivars | 141035 | species | *F. mandshurica* | Changshai.M |
| F2 | CS-004 | cultivars | 180059 | species | *F. vesca* | BailalSt94.13 |
| F2 | CS-008 | cultivars | 180217 | species | *F. vesca* | CFRA.371.001 |
| F2 | CS-009 | cultivars | 180606 | species | *F. vesca* | CFRA989.001 |
| F2 | CS-011 | cultivars | 190128 | species | *F. vesca* | F.Eibenstack |
| F2 | CS-012 | cultivars | 190172 | species | *F. vesca* | Korsika |
| F2 | CS-013 | cultivars | 190243 | species | *F. vesca* | Mt.Shasta |
| F2 | CS-015 | cultivars | 190255 | species | *F. vesca* | Tienshan.2 |
| F2 | CS-017 | cultivars | 190324 | species | *F. viridis* | Iran.1 |
| F2 | CS-021 | cultivars | 190349 | species | *F. viridis* | Plauen |
| F2 | CS-022 | cultivars | 190361 | species | *F. viridis* | St.4942B |
| F2 | CS-023 | cultivars | 190402 | species | *F.bifera* | Heimbergfurm.2 |
| F2 | CS-027 | cultivars | 190417 | species | *F.bifera* | Zuhzinger |
| F2 | CS-028 | cultivars | 201091 | species | *F.cascadensis* | PacificCrest.10 |
| F2 | CS-029 | cultivars | 201167 | species | *F.cascadensis* | WestBigLake4.4 |
| F2 | CS-030 | cultivars | 201357 | species | *F.chiloensis* | 2013.8 |
| F2 | CS-032 | cultivars | 201392 | species | *F.chiloensis* | Chile.1 |
| F2 | CS-033 | cultivars | 201401 | species | *F.chiloensis* | USA.1 |
| F2 | CS-034 | cultivars | 201409 | species | *F.iturupensis* | F.iturupensis.2020.4 |
| F2 | CS-035 | cultivars | 201419 | species | *F.moschata* | Profumata.di.Tortona |
| F2 | CS-036 | cultivars | 201438 | species | *F.moschata* | Rathenwald.2.1 |
| F2 | CS-037 | cultivars | 210017 | species | *F.nilgerrensis* | Dickoré1 |
| F2 | CS-038 | cultivars | 210035 | species | *F.nilgerrensis* | Mt.Leigong.1 |
| F2 | CS-039 | cultivars | 210056 | species | *F.nipponica* | St.05.6.1 |
| F2 | CS-040 | cultivars | 210087 | species | *F.nubicola* | Singalila |
| F2 | CS-041 | cultivars | 210096 | species | *F.virginiana* | Lac.Diabolo |
| F2 | CS-042 | cultivars | 210099 | species | *F.virginiana* | Montreal5.2.B |
| F2 | CS-043 | cultivars | 210103 | species | *F.virginiana* | Montreal5.4 |
| F2 | CS-045 | cultivars | 210140 | species | *F.virginiana* | Rocky.Mountains |
| F2 | CS-046 | cultivars | 210161 | species | *F.virginiana* | St.08.32 |
| F2 | CS-047 | cultivars | 210689 | species | *F.virginiana* | St.Anne1.8 |
| F2 | CS-048 | cultivars | 210697 |  |  |  |
| F2 | CS-049 | cultivars | 210706 |  |  |  |
| F2 | CS-052 | cultivars | 210726 |  |  |  |
| F2 | CS-053 | cultivars | 210727 |  |  |  |
| F2 | CS-055 | cultivars | 210738 |  |  |  |
| F2 | CS-057 | cultivars | 210745 |  |  |  |
| F2 | CS-059 | cultivars | 210757 |  |  |  |
| F2 | CS-061 | cultivars | 210764 |  |  |  |
| F2 | CS-062 | cultivars | 210789 |  |  |  |
| F2 | CS-063 | cultivars | 210795 |  |  |  |
| F2 | CS-064 | cultivars | 34-20-7C |  |  |  |
| F2 | CS-065 | cultivars | 90622 |  |  |  |
| F2 | CS-066 | cultivars | 94025 |  |  |  |
| F2 | CS-068 | cultivars | Asia |  |  |  |
| F2 | CS-069 | cultivars | Bravura |  |  |  |
| F2 | CS-071 | cultivars | Cerafine |  |  |  |
| F2 | CS-072 | cultivars | Clery |  |  |  |
| F2 | CS-073 | cultivars | Dahli |  |  |  |
| F2 | CS-074 | cultivars | Elsanta |  |  |  |
| F2 | CS-075 | cultivars | Elvie2 |  |  |  |
| F2 | CS-076 | cultivars | Faith |  |  |  |
| F2 | CS-077 | cultivars | Flair |  |  |  |
| F2 | CS-078 | cultivars | Florentina |  |  |  |
| F2 | CS-079 | cultivars | Lola |  |  |  |
| F2 | CS-081 | cultivars | Malling |  |  |  |
| F2 | CS-082 | cultivars | Marieka |  |  |  |
| F2 | CS-083 | cultivars | Renaissance |  |  |  |
| F2 | CS-084 | cultivars | Rendezvous |  |  |  |
| F2 | CS-086 | cultivars | Silvia |  |  |  |
| F2 | CS-087 | cultivars | Sonata |  |  |  |
| F2 | CS-088 | cultivars | Sonsation |  |  |  |
| F2 | CS-089 | cultivars | Vicontesse |  |  |  |
| F2 | CS-090 |  |  |  |  |  |
| F2 | CS-092 |  |  |  |  |  |
| F2 | CS-093 |  |  |  |  |  |
| F2 | CS-094 |  |  |  |  |  |
| F2 | CS-097 |  |  |  |  |  |
| F2 | CS-101 |  |  |  |  |  |
| F2 | CS-103 |  |  |  |  |  |
| F2 | CS-104 |  |  |  |  |  |
| F2 | P-90999 |  |  |  |  |  |
| F2 | SengaS |  |  |  |  |  |
| F2 | SengaS xF1 |  |  |  |  |  |
| F2 | USA1 |  |  |  |  |  |
