# Supplementary material for: Phenotyping 172 strawberry genotypes for water soaking reveals a close relationship with skin water permeance
Source: PeerJ. 2024 Aug 29;12:e17960. doi: 10.7717/peerj.17960 (PMC11366227; doi:10.7717/peerj.17960)
Supplement: Supplemental Information 3 — The variability was indexed by the coefficient of variation (CV). The collection of cultivars was sampled in the 2022 and the 2023 growing season, the two other collections in the 2022 season. [file peerj-12-17960-s003.docx]

**Table S2:**

**Mean, median and range between minimum and maximum, and variability for fruit mass, osmotic potential and water permeance of three collections of strawberries.**

The variability was indexed by the coefficient of variation (CV). The collection of cultivars was sampled in the 2022 and the 2023 growing season, the two other collections in the 2022 season.

| Collection | Season |  |  | Range | | CV (%) |
| --- | --- | --- | --- | --- | --- | --- |
|  |  | Mean | Median | Min | Max |  |
|  |  | Mass (g per fruit) | | | | |
| Cultivar | 2023 | 17.2 a | 17.2 | 8.1 | 24.1 | 21.9 |
|  | 2022 | 13.7 b | 13.3 | 4.3 | 24.7 | 29.3 |
| Species | 2022 | 1.6 d | 1.1 | 0.4 | 11.2 | 118.2 |
| F2 | 2022 | 2.3 c | 2.2 | 0.8 | 6.4 | 38.4 |
|  |  | Firmness (rating) | | | | |
| Cultivar | 2023 | 2.0 b | 2.0 | 1 | 3 | 38.1 |
|  | 2022 | 1.8 c | 2.0 | 1 | 3 | 40.7 |
| Species | 2022 | 1.8 c | 2.0 | 1 | 3 | 35.7 |
| F2 | 2022 | 2.2 a | 2.0 | 1 | 3 | 29.5 |
|  |  | Osmotic potential (MPa) | | | | |
| Cultivar | 2023 | -0.9 a | -0.9 | -1.1 | -0.6 | 15.5 |
|  | 2022 | -0.9 a | -0.9 | -1.1 | -0.6 | 15.4 |
| Species | 2022 | -1.2 b | -1.1 | -2.1 | -0.7 | 26.2 |
| F2 | 2022 | -1.2 b | -1.2 | -1.6 | -0.8 | 15.0 |
|  |  | Permeance (x 10^-6^ m s^-1^) | | | | |
| Cultivar | 2023 | 2.5 a | 2.5 | 1.0 | 5.1 | 38.0 |
|  | 2022 | 2.7 a | 2.6 | 1.0 | 6.2 | 40.4 |
| Species | 2022 | 2.6 a | 2.3 | 0.5 | 6.1 | 58.3 |
| F2 | 2022 | 1.9 b | 1.7 | 0.8 | 5.1 | 44.3 |

Means followed by the same letter do not differ. Mass, osmotic potential of fruit juice. and water permeance of the fruit skin were compared using analysis of variance and Tukey’s Studentized range test, P=0.05, fruit firmness scores using a non-parametric Pairwise Mann-Whitney U test at P=0.05
