# Supplementary material for: Phenotyping 172 strawberry genotypes for water soaking reveals a close relationship with skin water permeance
Source: PeerJ. 2024 Aug 29;12:e17960. doi: 10.7717/peerj.17960 (PMC11366227; doi:10.7717/peerj.17960)
Supplement: Supplemental Information 4 — Water soaking was indexed after 4 h of incubation in deionized water using a 5-point rating scale: score 0, no WS; score 1, <10% of the surface area water-soaked; score 2, 10–<35%; score 3, 35–60%; score 4, >60%. [file peerj-12-17960-s004.docx]

**Table S3:**

**Linear regression equations for relationship between the susceptibility to water-soaking and the log-transformed permeance of the fruit surface to water uptake in three collections of strawberries.**

Water soaking was indexed after 4 h of incubation in deionized water using a 5-point rating scale: score 0, no WS; score 1, <10% of the surface area water-soaked; score 2, 10–<35%; score 3, 35–60%; score 4, >60%.

| Collection | Season | Regression coefficients | | Coefficient of determination |
| --- | --- | --- | --- | --- |
|  |  | Slope ± SE | Intercept ± SE |  |
| Cultivar | 2023 | 2.51 ± 0.41 | 15.75 ± 2.33 | 0.56*** |
|  | 2022 | 2.95 ± 0.33 | 18.80 ± 1.84 | 0.57*** |
| Species | 2022 | 2.74 ± 0.30 | 18.04 ± 1.71 | 0.73*** |
| F2 | 2022 | 1.80 ± 0.31 | 12.17 ± 1.80 | 0.31*** |
| Grand mean |  | 2.41± 0.18 | 15.72± 1.05 | 0.46*** |

The collections comprised 31 (cultivars, 2023), 64 (cultivars, 2022), 32 (species, 2022) and 76 (F2, 2022) individual genotypes. The grand mean across all three populations in both years is based on 203 observations. Significance at P=0.001 indicated by ***.
