# Supplementary material for: Phenotyping 172 strawberry genotypes for water soaking reveals a close relationship with skin water permeance
Source: PeerJ. 2024 Aug 29;12:e17960. doi: 10.7717/peerj.17960 (PMC11366227; doi:10.7717/peerj.17960)
Supplement: Supplemental Information 5 — Firmness was indexed using a 3-point rating scale: score 1, firm; score 2, mid and 3, soft. The collections comprised 32 genotypes of different species sampled in 2022 (species) [file peerj-12-17960-s005.docx]

**Table S4:**

**Fruit characteristics of wild strawberry species from the Professor Staudt Collection.**

Firmness was indexed using a 3-point rating scale: score 1, firm; score 2, mid and 3, soft. The collections comprised 32 genotypes of different species sampled in 2022 (species)

| Species | Mass (g) | Firmness (rating) | Osmotic potential (MPa) |
| --- | --- | --- | --- |
| *F. × bifera* | 0.8±0.0 c* | 2.0±0.0 f | -1.4±0.1 b |
| *F. cascadensis* | 0.7±0.0 c | 2.0±0.0 f | -0.8±0.0 a |
| *F. chiloensis* | 5.8±0.7 a | 1.6±0.1 c | -1.2±0.0 cd |
| *F. iturupensis* | 2.4±0.3 bc | 2.0±0.0 bcf | -1.0±0.0 acd |
| *F. mandshurica* | 1.2±0.0 bc | 2.0±0.0 f | -1.2±0.0 bcd |
| *F. moschata* | 2.5±0.2 b | 3.0±0.0 a | -1.0±0.1 ad |
| *F. nilgerrensis* | 1.0±0.1 c | 2.0±0.0 f | -1.0±0.0 ad |
| *F. nipponica* | 0.6±0.1 c | 3.0±0.0 a | -0.7±0.0 a |
| *F. nubicola* | 1.7±0.1 bc | 3.0±0.0 a | -0.9±0.0 acd |
| *F. vesca* | 1.1±0.1 c | 2.6±0.1 e | -1.1±0.0 cd |
| *F. virginiana* | 0.9±0.0 c | 2.3±0.1 b | -1.2±0.0 bc |
| *F. viridis* | 1.5±0.1 bc | 1.0±0.0 d | -1.9±0.1 e |

*Mean separation within columns by Tukey’s test, P = 0.05.
