# Supplementary material for: Phenotyping 172 strawberry genotypes for water soaking reveals a close relationship with skin water permeance
Source: PeerJ. 2024 Aug 29;12:e17960. doi: 10.7717/peerj.17960 (PMC11366227; doi:10.7717/peerj.17960)
Supplement: Supplemental Information 6 — The genotypes were selected from the collection of cultivars and breeding clones based on their contrasting susceptibilities to water soaking (WS). [file peerj-12-17960-s006.docx]

**Table S5:**

**Masses of the cuticular membrane (CM), of dewaxed CM (DCM) and of wax of selected strawberry cultivars and breeding clones.**

The genotypes were selected from the collection of cultivars and breeding clones based on their contrasting susceptibilities to water soaking (WS).

| Year | Cultivar/Clone | Fruit mass (g) | Mass per unit area (g m^-2^) | | | Mass (mg per fruit) | | |
| --- | --- | --- | --- | --- | --- | --- | --- | --- |
|  |  |  | CM | DCM | wax | CM | DCM | wax |
| 2022 | 141033 | 7.7 | 0.81±0.1 | 0.66±0.1 | 0.15±0.03 | 1.56±0.2 | 1.27±0.19 | 0.29±0.05 |
| 2022 | 180059 | 16.8 | 0.53±0.03 | 0.47±0.02 | 0.06±0.01 | 1.71±0.09 | 1.51±0.07 | 0.19±0.04 |
| 2022 | 190243 | 11.5 | 0.63±0.03 | 0.57±0.03 | 0.06±0.01 | 1.58±0.07 | 1.43±0.06 | 0.15±0.03 |
| 2022 | 190349 | 14.4 | 0.57±0.03 | 0.46±0.03 | 0.11±0.02 | 1.66±0.09 | 1.34±0.1 | 0.32±0.04 |
| 2022 | 190361 | 21.1 | 0.53±0.1 | 0.46±0.1 | 0.07±0.01 | 1.98±0.38 | 1.73±0.36 | 0.25±0.04 |
| 2022 | 190402 | 13.8 | 0.7±0.03 | 0.59±0.03 | 0.11±0.01 | 1.98±0.09 | 1.66±0.09 | 0.32±0.04 |
| 2022 | 190417 | 18.8 | 0.5±0.04 | 0.42±0.04 | 0.08±0.01 | 1.75±0.14 | 1.45±0.13 | 0.29±0.04 |
| 2022 | 201091 | 14.8 | 0.55±0.03 | 0.41±0.02 | 0.14±0.02 | 1.62±0.08 | 1.21±0.05 | 0.41±0.05 |
| 2022 | 201167 | 13.1 | 0.61±0.04 | 0.52±0.02 | 0.09±0.03 | 1.68±0.1 | 1.43±0.04 | 0.24±0.09 |
| 2022 | 201409 | 13.5 | 0.54±0.02 | 0.47±0.02 | 0.07±0.02 | 1.51±0.05 | 1.31±0.04 | 0.2±0.05 |
| 2022 | 201419 | 12.8 | 0.47±0.01 | 0.39±0.01 | 0.08±0.01 | 1.26±0.04 | 1.06±0.04 | 0.2±0.04 |
| 2022 | 201438 | 15.4 | 0.47±0.01 | 0.38±0.02 | 0.09±0.01 | 1.42±0.04 | 1.16±0.05 | 0.27±0.03 |
| 2022 | 210017 | 14.3 | 0.44±0.03 | 0.4±0.03 | 0.04±0.01 | 1.29±0.08 | 1.16±0.08 | 0.12±0.03 |
| 2022 | 210087 | 8.9 | 0.51±0.03 | 0.41±0.02 | 0.1±0.02 | 1.08±0.06 | 0.88±0.04 | 0.2±0.04 |
| 2022 | 210706 | 9.3 | 0.61±0.04 | 0.53±0.04 | 0.08±0.01 | 1.35±0.09 | 1.17±0.08 | 0.17±0.03 |
| 2022 | 210726 | 15.7 | 0.5±0.02 | 0.41±0.01 | 0.09±0.02 | 1.54±0.07 | 1.27±0.04 | 0.28±0.05 |
| 2022 | 210764 | 18.4 | 0.66±0.04 | 0.53±0.03 | 0.13±0.01 | 2.25±0.12 | 1.81±0.1 | 0.44±0.05 |
| 2022 | 210795 | 15.3 | 0.5±0.02 | 0.42±0.01 | 0.08±0.01 | 1.52±0.06 | 1.28±0.04 | 0.25±0.04 |
| 2022 | 34-20-7C | 12.9 | 0.54±0.03 | 0.45±0.02 | 0.09±0.02 | 1.47±0.08 | 1.22±0.06 | 0.25±0.05 |
| 2022 | Dahli | 18.9 | 0.50±0.06 | 0.41±0.03 | 0.10±0.05 | 1.75±0.22 | 1.44±0.12 | 0.35±0.18 |
| 2022 | Elvie2 | 11.8 | 0.59±0.05 | 0.51±0.04 | 0.08±0.02 | 1.51±0.14 | 1.30±0.10 | 0.21±0.05 |
| 2022 | Lola | 18.6 | 0.54±0.02 | 0.43±0.02 | 0.11±0.01 | 1.87±0.08 | 1.48±0.06 | 0.39±0.05 |
| 2022 | Malling-champion | 12.5 | 0.58±0.08 | 0.49±0.05 | 0.10±0.03 | 1.54±0.2 | 1.29±0.12 | 0.25±0.09 |
| 2022 | Renaissance | 10.5 | 0.77±0.16 | 0.73±0.16 | 0.04±0.01 | 1.82±0.37 | 1.73±0.37 | 0.09±0.03 |
| 2022 | rendezvous | 15.1 | 0.58±0.03 | 0.45±0.03 | 0.13±0.02 | 1.74±0.1 | 1.35±0.10 | 0.38±0.06 |
| 2022 | Silvia | 12.2 | 0.51±0.03 | 0.45±0.03 | 0.06±0.02 | 1.33±0.07 | 1.18±0.07 | 0.15±0.05 |
| 2022 | Sonsation | 14.5 | 0.42±0.05 | 0.33±0.02 | 0.09±0.03 | 1.23±0.14 | 0.97±0.07 | 0.26±0.09 |
| 2023 | 190128 | 13.2 | 0.60±0.04 | 0.51±0.03 | 0.08±0.01 | 1.66±0.1 | 1.40±0.09 | 0.21±0.03 |
| 2023 | 190349 | 25.1 | 0.53±0.03 | 0.36±0.01 | 0.16±0.02 | 2.2±0.11 | 1.51±0.04 | 0.69±0.10 |
| 2023 | 201409 | 13.3 | 0.48±0.02 | 0.42±0.02 | 0.08±0.02 | 1.33±0.06 | 1.17±0.06 | 0.21±0.04 |
| 2023 | 201419 | 33.2 | 0.59±0.04 | 0.38±0.01 | 0.21±0.04 | 2.97±0.22 | 1.91±0.05 | 1.06±0.21 |
| 2023 | 201438 | 29.7 | 0.47±0.02 | 0.35±0.01 | 0.11±0.02 | 2.19±0.11 | 1.64±0.05 | 0.53±0.11 |
| 2023 | 210706 | 11.3 | 0.57±0.02 | 0.43±0.01 | 0.14±0.01 | 1.42±0.04 | 1.07±0.02 | 0.35±0.03 |
| 2023 | Asia | 19.3 | 0.57±0.01 | 0.40±0.01 | 0.17±0.01 | 2±0.04 | 1.40±0.03 | 0.61±0.05 |
| 2023 | Clery | 16.9 | 0.48±0.02 | 0.38±0.01 | 0.10±0.02 | 1.56±0.06 | 1.23±0.04 | 0.33±0.06 |
| 2023 | Florentina | 17.7 | 0.52±0.03 | 0.40±0.02 | 0.12±0.02 | 1.73±0.11 | 1.34±0.06 | 0.40±0.08 |
| 2023 | Lola | 22.9 | 0.50±0.04 | 0.39±0.04 | 0.12±0.02 | 1.98±0.15 | 1.52±0.15 | 0.46±0.06 |
