# Supplementary material for: Phenotyping 172 strawberry genotypes for water soaking reveals a close relationship with skin water permeance
Source: PeerJ. 2024 Aug 29;12:e17960. doi: 10.7717/peerj.17960 (PMC11366227; doi:10.7717/peerj.17960)
Supplement: Supplemental Information 7 — The genotypes were selected from the collection of cultivars and breeding clones based on their contrasting susceptibilities to water soaking (WS) and their achene position relative to the surface of the receptacle. The genotypes were scored using a three-step rating scheme, where score 1 represented achenes sunken, score 2 achenes level, and score 3 achenes protruding. [file peerj-12-17960-s007.docx]

**Table S6:**

**Characteristics of the achene depression of selected strawberry cultivars and clones.**

The genotypes were selected from the collection of cultivars and breeding clones based on their contrasting susceptibilities to water soaking (WS) and their achene position relative to the surface of the receptacle. The genotypes were scored using a three-step rating scheme, where score 1 represented achenes sunken, score 2 achenes level, and score 3 achenes protruding.

| Genotype | Achenes position  (score) | Depth of achene depression (µm) | Surface area of achene depression (mm^2^) |
| --- | --- | --- | --- |
| Lola | 2 | 922.8±48.9 | 10.3±0.5 |
| 201409 | 3 | 486.5±59.0 | 7.1±0.4 |
| Florentina | 3 | 594.2±55.1 | 8.0±0.5 |
| 190349 | 2 | 843.8±76.3 | 11.4±0.8 |
| Clery | 2 | 861.4±57.9 | 9.4±0.6 |
| Asia | 2 | 1144.4±82.4 | 11.6±0.4 |
| 201438 | 2 | 613.5±16.4 | 7.7±0.4 |
| 201419 | 2 | 790.1±34.3 | 10.2±0.5 |
| 190128 | 2 | 947.0±53.1 | 11.8±0.5 |
| 210706 | 2 | 854.9±82.6 | 10.1±0.7 |
| 190243 | 1 | 1592.9±89.4 | 17.9±0.9 |
| 210757 | 1 | 1156.9±72.2 | 12.3±0.8 |
| Elsanta | 3 | 580.0±52.5 | 7.1±0.4 |
| Elvie2 | 1 | 1127.3±81.6 | 11.3±0.5 |
