# Supplementary material for: Phenotyping 172 strawberry genotypes for water soaking reveals a close relationship with skin water permeance
Source: PeerJ. 2024 Aug 29;12:e17960. doi: 10.7717/peerj.17960 (PMC11366227; doi:10.7717/peerj.17960)
Supplement: Supplemental Information 8 — Water soaking (WS) was indexed after 4 h of incubation in deionized water using a 5-point rating scale: score 0, no WS; score 1, <10% of the surface area water-soaked; score 2, 10–35%; score 3, 35–60%; score 4, >60%. [file peerj-12-17960-s008.docx]

**Table S7:**

**Coefficients of correlation among different parameters from 31 genotypes of the cultivar collection in two years of production.**

Water soaking (WS) was indexed after 4 h of incubation in deionized water using a 5-point rating scale: score 0, no WS; score 1, <10% of the surface area water-soaked; score 2, 10–35%; score 3, 35–60%; score 4, >60%.

| Parameter | Year | WS at 4 h | Time lag | Water uptake characteristics | | | |
| --- | --- | --- | --- | --- | --- | --- | --- |
|  |  | (rating) | (h) | Flow  (mg h^-1^) | Flux density  (kg m^-2^s^-1^) | Water permeance  (x10^-6^ m s^-1^) | Log permeance |
| Osmotic potential (MPa) | 2022 | -0.03^ns^ | 0.17 ^ns^ | -0.14 ^ns^ | -0.19 ^ns^ | 0.14 ^ns^ | 0.14 ^ns^ |
|  | 2023 | -0.28 ^ns^ | 0.35 ^ns^ | -0.50** | -0.51** | -0.24 ^ns^ | -0.19 ^ns^ |
| WS  (rating) | 2022 |  | -0.75*** | 0.57*** | 0.74*** | 0.72*** | 0.74*** |
|  | 2023 |  | -0.71*** | 0.64*** | 0.77*** | 0.78*** | 0.75*** |
| Time lag (h) | 2022 |  |  | -0.43* | -0.41* | -0.35^ns^ | -0.43* |
|  | 2023 |  |  | -0.60*** | -0.65*** | -0.61*** | -0.59*** |
| Flow rate  (mg h^-1^) | 2022 |  |  |  | 0.85*** | 0.82*** | 0.81*** |
|  | 2023 |  |  |  | 0.94*** | 0.89*** | 0.84*** |
| Flux density  (kg m^-2^s^-1^) | 2022 |  |  |  |  | 0.94** | 0.92** |
|  | 2023 |  |  |  |  | 0.95*** | 0.90*** |
| Permeance  (x10^-6^m s^-1^) | 2022 |  |  |  |  |  | 0.97*** |
|  | 2023 |  |  |  |  |  | 0.96*** |

Significance of levels at P=0.001, 0.01, and 0.05 indicated by ***, ** and *. ^ns^ non-significant.
