# Supplementary figures and images for: Phenotyping 172 strawberry genotypes for water soaking reveals a close relationship with skin water permeance
Source: PeerJ. 2024 Aug 29;12:e17960. doi: 10.7717/peerj.17960 (PMC11366227; doi:10.7717/peerj.17960)

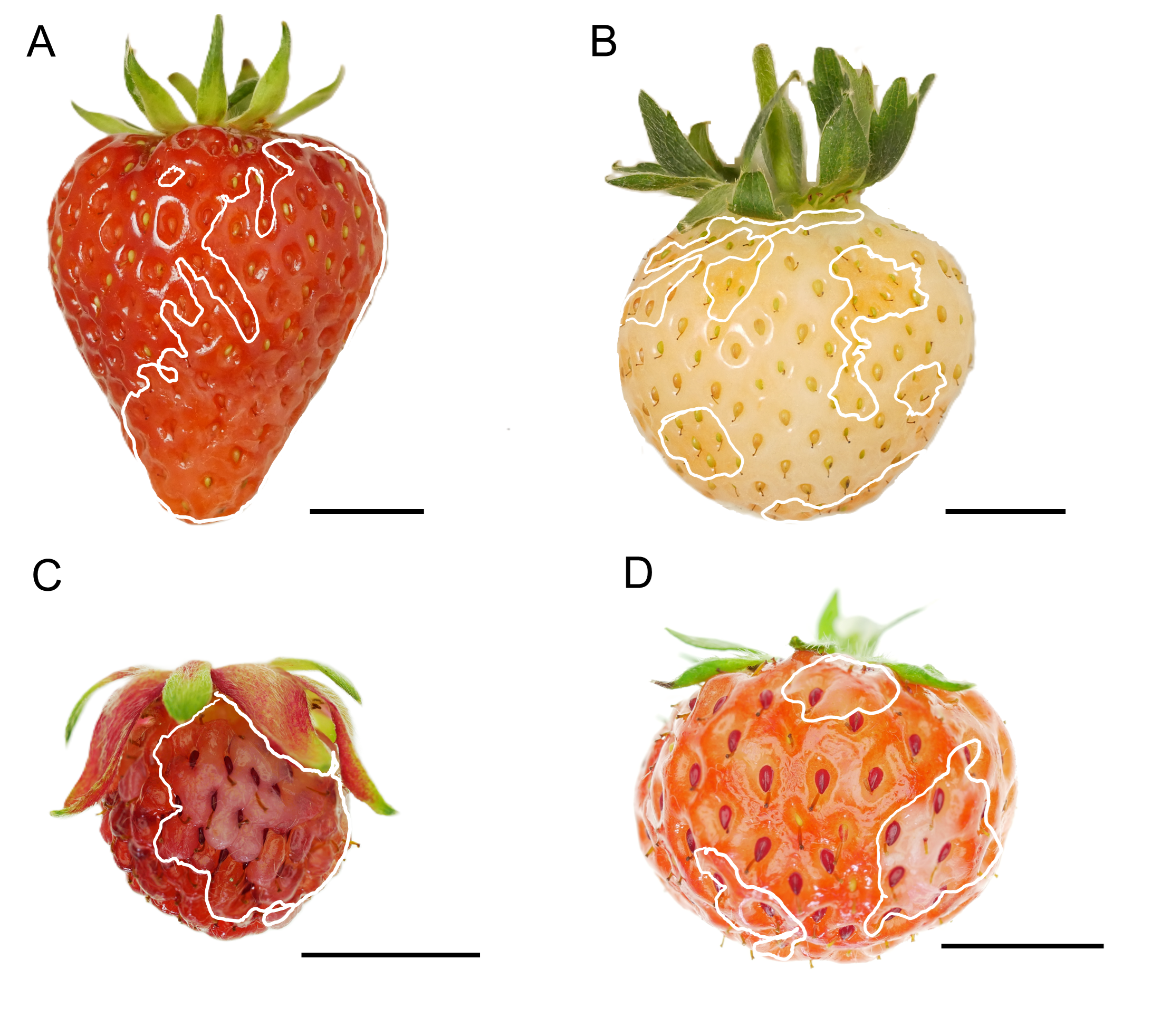

Supplement: Supplemental Information 9 — (A,B) Clones ‘141035’ (A) and ‘190172’ (B) of the cultivar collection. (C) Genotype ‘USA 1’ of Fragaria chiloensis of the species collection, and (D) genotype ‘CS-027’ of the segregating F2 population. The white line traces the perimeter of the water-soaked area. Scale bar scale in all images = 1 cm. [file peerj-12-17960-s009.png]
